# Infant birth weight estimation and low birth weight classification in United Arab Emirates using machine learning algorithms

**DOI:** 10.1038/s41598-022-14393-6

**Published:** 2022-07-15

**Authors:** Wasif Khan, Nazar Zaki, Mohammad M. Masud, Amir Ahmad, Luqman Ali, Nasloon Ali, Luai A. Ahmed

**Affiliations:** 1 Department of Computer Science and Software Engineering, College of Information Technology, United Arab Emirates University, 15551 Al Ain, United Arab Emirates; 2 Big Data Analytics Center, United Arab Emirates University, 15551 Al Ain, United Arab Emirates; 3 Department of Information Systems and Security, College of Information Technology, United Arab Emirates University, 15551 Al Ain, United Arab Emirates; 4 Institute of Public Health, College of Medicine and Health Sciences, United Arab Emirates University, P.O. Box 15551, Al Ain, United Arab Emirates; 5 Zayed Centre for Health Sciences, United Arab Emirates University, P.O. Box 17666, Al Ain, United Arab Emirates

**Keywords:** Health care, Risk factors

## Abstract

Accurate prediction of a newborn’s birth weight (BW) is a crucial determinant to evaluate the newborn’s health and safety. Infants with low BW (LBW) are at a higher risk of serious short- and long-term health outcomes. Over the past decade, machine learning (ML) techniques have shown a successful breakthrough in the field of medical diagnostics. Various automated systems have been proposed that use maternal features for LBW prediction. However, each proposed system uses different maternal features for LBW classification and estimation. Therefore, this paper provides a detailed setup for BW estimation and LBW classification. Multiple subsets of features were combined to perform predictions with and without feature selection techniques. Furthermore, the synthetic minority oversampling technique was employed to oversample the minority class. The performance of 30 ML algorithms was evaluated for both infant BW estimation and LBW classification. Experiments were performed on a self-created dataset with 88 features. The dataset was obtained from 821 women from three hospitals in the United Arab Emirates. Different performance metrics, such as mean absolute error and mean absolute percent error, were used for BW estimation. Accuracy, precision, recall, F-scores, and confusion matrices were used for LBW classification. Extensive experiments performed using five-folds cross validation show that the best weight estimation was obtained using Random Forest algorithm with mean absolute error of 294.53 g while the best classification performance was obtained using Logistic Regression with SMOTE oversampling techniques that achieved accuracy, precision, recall and F1 score of 90.24%, 87.6%, 90.2% and 0.89, respectively. The results also suggest that features such as diabetes, hypertension, and gestational age, play a vital role in LBW classification.

## Introduction

Birth weight (BW) plays an important role in the survival and health of newborns, and accurate BW prediction will help healthcare practitioners make timely decisions. Newborns with a BW of ≤ 2500 g are considered as low BW (LBW) infants. Low BW in infants can occur because of various reasons such as maternal diet, close pregnancy intervals, infections, high parity, preterm delivery, and socioeconomic factors. Compared with normal BW infants, LBW infants are at a higher risk of perinatal death at a ratio of 8:1^1^. Moreover, LBW infants have a greater chance of having serious development problems such as low intelligence quotient (IQ), mental retardation, visual and hearing impairment, neonatal hypothermia, neonatal hypoglycemia, long-term disabilities, and premature death^2,3^. Detecting LBW infants before birth may substantially reduce such risks compared with identifying such infants after birth. Therefore, accurate and timely diagnosis of LBW infants is essential for medical practitioners to reduce the risk factors for mothers and infants by providing appropriate interventions and improving the overall prognosis.

Recently, to provide medical practitioners with better prognosis and diagnosis support, machine learning (ML) algorithms have become a standard choice for professional medical applications such as BW estimation and classification^1,3^. However, there are several challenges associated with creating such ML-based systems. ML-based systems require quality data^4^ for training and evaluation; however, creating such a high quality dataset is difficult because most medical data are not publicly available owing to copyright and privacy laws. Furthermore, some records in these datasets contain missing records, which is quite common in medical related data^5,6^ and impacts the overall performance of an ML-based system.

Datasets with high dimensions present another challenge for data mining and classification tasks. Typically, high-dimensional datasets include a large number of ineffective or unnecessary variables that can negatively affect the ML model's performance. To address this problem and improve the overall performance, feature selection algorithms are used to select relevant and important features from the dataset^7^. Several techniques are reported in literature^7,8^ that select an optimal feature set for adequately representing the dataset to improve overall performance. The datasets used in current LBW classification studies are highly class imbalanced, i.e., the number of data points available for different classes differs. Class imbalance considerably degrades the efficiency of a classification system. Traditionally, to address this issue, the minority class is oversampled by duplicating the randomly selected samples and the majority class is undersampled. The synthetic minority oversampling technique (SMOTE)^9^ is a well-known data balancing method, which oversamples the minority class by creating synthesized samples based on the similarities between pairs of the existing minority instances^4,9^. The SMOTE is a simple yet efficient algorithm that outperforms state-of-the-art generative adversarial networks (GANs)^10^. Therefore, in this study, SMOTE is adopted for data balancing. LBW and normal birthweight (NBW) can be classified based on the features provided to various classifiers, such as support vector machines (SVM), logistic regression (LR), naïve Bayes (NB), and random forest (RF). Previous studies have evaluated the performance of multiple ML models using heterogeneous datasets and different performance metrics. However, to the best of our knowledge, no study has provided a detailed evaluation of multiple ML models using multiple performance metrics on several subsets of features.

The primary objective of this paper is to evaluate the performance of 30 ML models for BW estimation and LBW classification using different subsets of data obtained from mothers during their pregnancy in three hospitals of the United Arab Emirates (UAE). The dataset used in this study contains data from 821 Emirati (UAE nationality) women. This dataset uses features similar to those used in previous studies^11–16^ (herein, each dataset is called a subset); all the features are combined to create one large dataset that contains six subsets.

The primary contributions of this paper are as follows.

1. We proposed a self-created dataset that contained features similar to those used by Hussain et al.^11^, Faruk et al.^12^, Kuhle et al.^13^, Senthilkumar et al.^14^, Loreto et al.^15^, and Kader et al.^16^. The created dataset contained 88 features, including infant BW as a target label. We refer this dataset as original dataset.
2. The performance of 30 ML models was evaluated. The evaluation results were used for BW estimation and LBW classification.
3. Multiple experiments were performed on all features and reduced features. In addition, feature selection was employed on the entire dataset.
4. To handle the class imbalance problem, we used the SMOTE method to oversample the minority class with four different oversampling ratios. We used the SMOTE because it is computationally less complex and outperforms well known state-of-the art methods, such as GANs^10^.
5. We provided recommendations and suggestions for future work to help researchers select the most effective and efficient regression and classification methods. Furthermore, this study provided a baseline for researchers working in the medical domain, particularly in the UAE.

The remainder of this paper is organized as follows. The second section discusses previous work related to BW prediction and classification. The proposed methodology is described in third section, and the experimental results are presented in fourth section. The problems associated with LBW infants and our experiments as well as our experimental results are discussed in fifth section followed by conclusion in last section.

## Related work

Most previous studies that investigate infant BW estimation and LBW classification employ ML algorithms. Feng et al.^17^ proposed an SVM-based classification model built using a dynamic Bayesian network (DBN) for fetal weight estimation from ultrasound parameters. The authors used a dataset collected from 7875 women with a singleton fetus in West China Secondary Hospital. They used SMOTE for data balancing because only 190 (2.41%) of the 7875 instances were from the LBW class. Trujillo et al.^18^ used a dataset obtained from the National Institute of Perinatology of Mexico which contained data from 250 women and included 23 features to estimate BW. Senthilkumar et al.^14^ compared the performance of six ML algorithms (NB, RF, neural network (NN), Decision Tree (DT), SVM, and LR) for LBW predictions. They used a dataset with 11 features obtained from 189 pregnant women (130 NBW babies and 59 LBW babies). A similar study conducted by Borson et al.^19^ used a dataset of 448 instances with 10 features for LBW classification. Faruk et al.^12^ applied LR and RF to LBW data for their prediction and classification. They used a dataset obtained from the 2007–2012 Indonesian Demographic and Health Surveys. The dataset contained data from 12,055 women aged from 15 to 49 years which contains 8 features.

Yarlapati et al.^20^ used a Bayes minimum error rate classifier to classify LBW and normal BW. The authors collected a dataset from Indian health camps between July 2015 and October 2016. The dataset contained data from 101 patient reports with 18 features. Al Habashneh et al.^21^ used ROC curve analysis for investigating preterm births and LBW infants using maternal data obtained from 227 pregnant Jordanian women (≤ 20 weeks of gestation). Ahmadi et al.^22^ applied LR and RF to predict LBW (< 2500 g) on a dataset obtained from the Milad Hospital in Iran. The data were obtained from 600 pregnant women; however, only 9.5% of the cases were LBW. Desiani et al.^1^ applied an NB classifier to maternal data for predicting the weight of infants delivered by hypertensive and nonhypertensive mothers. Their dataset included the data of 219 patients from Muhammadiyah Hospital Palembang in Indonesia.

Lu et al.^26^ proposed a genetic algorithm (GA) based ensemble learning model to estimate fetal weight at any gestational age. The authors used a dataset that was obtained from a hospital in Shenzhen, China, and contained electronic health records of 4,212 pregnant women with 14 features. Kuhle et al.^13^ compared the performance of an LR model with those of other machine learning algorithms (RF, DT, elastic Net, NNet, and GradientBoosting) for small for gestational age (SGA), appropriate GA (AGA), and large gestational age (LGA) prediction using data from 30,705 pregnant women in the Canadian province of Nova Scotia. Li et al.^3^ evaluated different ML approaches for SGA using an SGA dataset. The dataset was collected from the Prepregnancy Program in China between 2010 and 2013. The data comprised 215,568 records of parent pregnancy examinations with 371 features. The authors selected a total of 85,161 records that were divided into SGA and nonSGA cases. Akhtar et al.^6^ also used the Prepregnancy Program’s dataset and employed ML techniques to predict LGA, i.e., newborn’s weight above the 90^th^ percentile at the same gestational age. The authors selected 102,219 infants as LGA and 189,342 as nonLGA for their experiments. Another study by Akhtar et al.^23^ proposed feature selection followed by classification. Grid search*-*based recursive feature elimination with cross-validation (RFECV) was used for feature selection followed by IG to rank the features subset. They used 26,226 records out of the 215,568 records from the Prepregnancy Program and labeled them as LGA. The remaining 189,342 records were labeled as nonLGA. An ensemble stacked classifier was used to minimize the generalization error.

Kumar et al.^24^ used polycyclic aromatic hydrocarbon (PAH) and sociodemographic features to predict the LBW of newborns. They collected the data of 120 women who delivered NBW babies and 55 women who delivered LBW babies. The data came from Assam Medical College in India. Hussain et al.^11^ proposed two ML techniques: RF and Gaussian naïve Bayes to classify LBW and NBW from a self-created dataset that contained 445 instances and 18 features. The dataset was collected from two government centers in India and included the data of 445 pregnant women with 18 features. Akbulut et al.^25^ proposed an artificial intelligence-based system to predict the fetal anomaly status (fetal health status) based on maternal clinical data. The authors collected a dataset of 96 pregnant women that contained a maternal questionnaire and a detailed evaluation by three clinicians from RadyoEmar Imaging Center, a medical diagnostic imaging center in Istanbul, Turkey. Loreto et al.^15^ evaluated the performance of six ML algorithms for LBW classification, i.e., RF, adaptive boosting (AdaBoost), NB, KNN, SVM, and DT. A dataset of 2,328 instances was used. The data were obtained from the obstetrics services provided by a Portuguese hospital. The dataset was imbalanced; therefore, an oversampling technique was applied. The summary of the literature done for BW estimation and LBW classification is represented in Table 1.

**Table 1:** Work related to LBW classification.

References	Problem and approach	Approach	Prepro Tech algorithms/method	ML models	Performance
Feng et al. 2019^17^	Fetal weight estimation	Estimation and classification	SMOTE for data balancing	SVM classification, DBN for weight estimation	MAE of 198.55 g ± 158 g, MAPE of 6.09 ± 5.06%
Kuhle et al. 2018^13^	SGA, AGA, and LGA	Classification	Data balancing^11^	LR, EN, CT, RF, GB, and NN	An AUC of 0.6–0.70 for primiparous women, while an AUC of 0.7–0.8 for multiparous women for SGA and LGA prediction
Sebthilkumar et al. 2015^14^	LBW prediction	Classification	–*	NB, RF, NN, DT, SVM, and LR	DT classifier with an accuracy of 0.899, a sensitivity of 0.97 and a specificity and AUC of 0.72 and 0.93, respectively
Borson et al. 2020^19^	LBW prediction	Classification	Redundant feature elimination, elimination of unique features, missing values handling, attribute transformation	LR, NB, KNN, and MLP	The best accuracy of 81.67% was achieved by SVM and MLP
Loreto et al. 2019^15^	LBW prediction	Classification	Elimination of records with missing data, normalization, oversampling techniques	KNN, Tree, NB, RF, SVM, and AdaBoost	AdaBoost classifier showed better classification performance with an accuracy of 98% and a sensitivity and specificity of 0.91 and 0.99, respectively
Kumar et al. 2020^24^	LBW prediction from PAH	Classification	Women with existing health conditions, such as HIV and diabetes, were excluded	SVM, AdaBoost, NB	The SVM classifier achieved an accuracy of 81.21% and a sensitivity and specificity of 0.84 and 0.74, respectively
Anisha et al. 2017^20^	LBW prediction	Classification	Eliminate significant missing values	Feature ranking using RF and XGBoost, and NB-based minimum error rate classifier	Bayes Minimum Error was used for classification that achieved an accuracy of 0.967 and a sensitivity and specificity of 1.0 and 0.85, respectively
Faruk et al. 2018^12^	LBW prediction	Prediction and classification	Missing records were deleted	RF and LR	RF achieved 93% accuracy
Akhtar et al. 2020^6^	LGA	Classification	Variable discretization, removing instances that had more than 30% missing values. missing value with less than 30 were replaced with mean and mode	Feature determination, SVM, RF, LR, and NB	A precision of 0.84 and an AUC of 0.72 with top 30 using SVM
Akhtar et al. 2019^23^	LGA	Classification	IG, Grid Search based RFECVa + IG	SVM and DT	An accuracy of 92% using an SVM classifier with a linear kernel precision of 0.92, a recall of 0.87 and a specificity of 0.95
Al Habashneh et al. 2012^21^	LBW and PB	ROC analysis	–	ROC analysis	For LBW, an AUC of 0.81 LBW using CAL and a sensitivity and specificity of 0.81 and 0.70, respectively, for CAL with a cutoff value of 0.42 mm
Li et al. 2020^3^	SGA	Prediction	Feature discretization, missing value as a separate value of 0	SVM, RF, LR, and Sparse LR	Sparse LR performed well by achieving an AUC of 0.817
Desiani et al. 2019^1^	Birthweight in hypertensive mothers	Classification	Removing variables with ambiguous data	NB classifier	An accuracy of 81.25% and a precision and recall of 1.00 and 0.75, respectively, for LBW
Ahmadi et al. 2017^22^	LBW prediction	Classification	–	RF and LR	An accuracy of 95% with 97% specificity and 72% sensitivity using RF
Hussain et al. 2020^11^	LBW	Classification	Missing values were replaced with average of nearby cells	RF and Gaussian NB	An accuracy of RF is 100% with the precision, recall, and F1 score of 1.0
Lu et al. 2019^26^	Fetal weight estimation	Estimation	Normalization	Ensemble of RF, XGBoost, and lightGBM	An MRE of 7% with an accuracy of 64.3%
Akbulut et. al. 2018^25^	Health status (normal or pathological)	Classification	–	AP, BDT, BPM, DF, LR, SVM, and NN	Web and mobile application development of 89.5% was achieved using decision forest
Trujillo et al. 2020^18^	BW estimation	Estimation	–	SVR	SVR with RBF kernel achieved better accuracy with an MAE of 287.60 ± 195.86 (g) and an MPE of 0.364% ± 11.95%

## Proposed methodology

**Figure 1:**
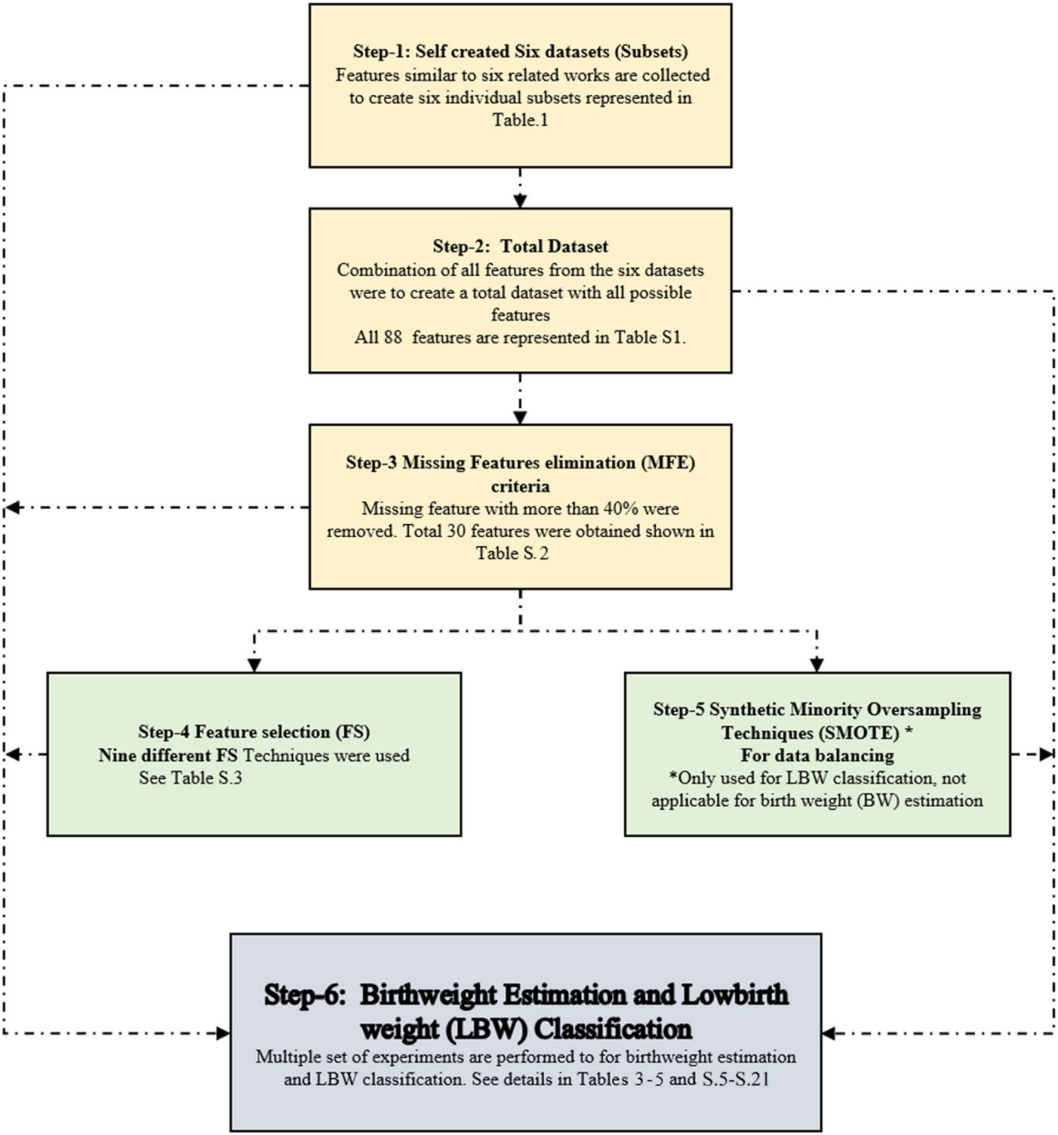
Proposed ML framework for infant weight estimation and LBW classification.

A flowchart of the proposed methodology is shown in Fig. 1, which indicates that the first six different subsets of features are created followed by a combination of all the subsets to create a full dataset (D(all features)). Notably, the features with greater than 40% missing or not applicable values were removed. Feature selection techniques were then employed to select the most appropriate features for BW estimation and LBW classification. Furthermore, the dataset used in this study is highly class imbalanced; therefore, SMOTE was used for data balancing with multiple oversampling ratios for LBW classification. Experiments were performed on each module shown in Fig. 1. The results of the proposed models were evaluated and analyzed using various performance metrics, which are explained in the last module. Each module is described in detail as the following.

### Data collection and data preprocessing

The data used in this study were obtained from ongoing birth cohort in the UAE. Details about the study can be found elsewhere in the literature^27^. Medical data were extracted from the medical records from the three recruiting hospitals at the time of delivery which included the reproductive history. We obtained a list of features from the pregnant women that were used in the current study, such as the features used by authors in Table 2. We performed experiments on each subset of features and then combined all the features to demonstrate the effect of these features on the overall BW estimation and LBW classification performances. Each subset of features description is presented in Table S1.

**Table 2:** Features used in this study (each subset represents the features used in previous LBW classification studies).

Dataset name and authors	Classification/regression task	Total features	Feature names that were used in this study	Feature that were not available to us
Subset-1; Hussain et al. 2020^11^	LBW classification	445 samples with 18 features. Binary classification	Socioeconomic condition, age, height, BGroup, parity, antenatal check, initial weight of mother, final weight of mother (Last ANC), initial systolic blood pressure, initial diastolic blood pressure, final systolic blood pressure (Last ANC), final diastolic blood pressure (last ANC), initial hemoglobin level, final hemoglobin level (Last ANC), blood sugar (Random), TermPreterm Term: 37–40 weeks, preterm: < 37 weeks, sex, and weight	Socioeconomic condition, antenatal check, and blood sugar (random)
Subset-2; Faruk et al. 2018^12^	Prediction and classification	9 features including BW	Place of residence, time zone, wealth index, mother’s education, father’s education, age of mother, job of mother, and the number of children	Time zone, wealth index, and father’s job
Subset-3; Khule et al. 2018^13^	SGA, AGA, and LGA classification	30,705 pregnancy samples with complete information of all variables **23 features** (Sociodemographic, pregnancy risk factors, past pregnancy history, current pregnancy)	Maternal age, common law/married, area-level income quintiles, urban residence, smoking before pregnancy, prepregnancy BMI [m/kg^2^], pre-existing hypertension, pre-existing diabetes, previous gestational diabetes, previous child with BW < 2500 g, previous child with BW > 4080 g, previous caesarean section, previous preterm delivery < 29 weeks, previous preterm delivery 29–32 weeks, previous preterm delivery 33–36 weeks, previous death of neonate ≥ 500 g, fetal male sex, weight gain at 26 weeks [kg], smoking during pregnancy, substance use during pregnancy, gestational diabetes, pregnancy-induced hypertension, and psychiatric disorder	Area-level income quintiles, urban residence, weight gain at 26 weeks [kg], smoking during pregnancy, substance use in pregnancy, pregnancy-induced hypertension, and psychiatric disorder
Subset-4; Sethilkumar et al. 2015^14^	LBW classification	11 features	years (age), the weight of the mother at her last menstrual period (LWT), the number of physician visits during the first trimester of pregnancy (FTV), race (RACE), lifestyle information, e.g., smoking (smoke), history of previous preterm delivery (PTL), the presence of uterine irritability (UI), and hypertension (HT)	Race and UI
Subset-5; Loreto et al. 2019^15^	LBW classification	9 features and 2328 instances	Multiplicity (whether the gestation is multiple) smoker, hypertension, diabetes, age, BMI, gestational age, fetus sex, and fetus weight	Multiplicity (when gestation is multiple)
Subset-6; Kader and Nirmala 2014^16^	LBW	20,946 instances, 11 features	Sex, wealth status, caste/tribe, age, education, BMI, stature, anemia level, interpregnancy interval, antenatal visits, and living place	Wealth status, caste/tribe, anemia level, and living place

The combined dataset contains a total of 821 instances, and each instance contains 88 features including BW as a target variable. Table S1 describes the features (original features we obtained: **D1**) considered in this study, along with their description and missingness ratio. Furthermore, features with greater than 40% missing values or values which were not applicable were removed, and we refer to this set of features as **D2**. We refer to the removal of features with more than 40% missing values as the missing features elimination (MFE) criterion, and the dataset obtained after MFE is referred as D2. The dataset obtained by combining all the features and after employing MFE criteria contains 30 features (Table S2). Experiments were performed on the D1 and D2 features to observe the impact of missingness in the data. Moreover, each subset was evaluated on the basis of the original features (D1) and the features obtained after removing the missing values (D2).

Furthermore, if a dataset contains too many features, the computational cost may increase if all features are selected. Note that removing features may eliminate important features and degrade the performance of an ML algorithm. Therefore, to select an optimal feature set to improve performance, we employed various feature selection techniques^28–33^ (Table S3) for the BW estimation and LBW classification. The frequency of each feature selected by each FS algorithm was calculated and approximately half of the features that appeared in at least 40% were selected.

Another serious issue that occurs with the medical datasets for classification is class imbalance, which affects the performance of the ML algorithms, can lead to results that are biased toward the majority class, and even the misclassification of all minority instances^26^. The dataset used in this study is also highly class imbalanced at a ratio of 1:8, i.e., only 89 samples belonged to the minority class (i.e., LBW) and 732 samples belonged to the normal class. An imbalanced dataset seriously degrades the performance of the ML model^4^; therefore, we oversampled the minority class using SMOTE^7^ to balance the dataset. SMOTE is less computationally complex compared to common state-of-the-art methods such as GANs. SMOTE was applied to the entire dataset using multiple balancing ratio such as the minority class was oversampled by 50%, 100%, 300% and totally balanced dataset. The oversampled data were only included in the training set, and no artificial samples were used in the testing set.

### Machine learning algorithms

The final feature vector obtained from the preprocessing step will be used for predicting the instances where the BW estimation and classification will be conducted on the basis of feature’s relevance using multiple ML models. The performance evaluation of different ML models^35–48^ used in this study is presented in Table S4.

### Performance metrics

Multiple performance metrics were used to evaluate the results obtained from each algorithm. For example, the weight estimation MAE and MAPE were used^17,18,49^. Similarly, for LBW classification, several performance metrics such as accuracy, precision, recall, F-score, and confusion matrix were considered^4^.

## Experimental results

In this study, the experiments were conducted using Weka on an Intel® Core™ i7-8700 CPU@3.200 GHz 3.19 GHz desktop system with 8.0 GB RAM.

### BW estimation

For BW estimation, the ten-fold cross validation technique was employed to obtain optimal predictions. In this study, the experiments were conducted using each subset and the combined features and each experiment was performed using D1 and D2. Table S5 shows the performance evaluations of multiple ML models using the features employed by Hussain et al.^11^ (Subset-1). The results show that the best performance was achieved using RF with a MAE value of 349.96 and an MAPE value of 13.91% when all 27 features (D1) were used. However, the results obtained using D2 show that when employing 18 features, the best result was achieved using SMOregression with a MAE value of 308.98. Table S6 shows the results obtained for Subset-2 (features used by Faruk et al.^12^), which contains 5 features. As all the features in Subset-1 contain less than 40% missing values; hence, the MFE criterion was not applied, and experiments were performed on the complete subset of features (D1). The results show that the SVR with epsilon performed well compared with all the other algorithms, showing a MAE value of 361.74 and an MAPE value of 14.57%. Similarly, the results for Subset-3 presented in Table S7 indicate that RF performed well with the D1 features, affording the MAE and MAPE values of 345.08 and 13.76%, respectively. The worst performance was obtained using the MLP method on all the three subsets.

The experimental results obtained for feature Subset-4 are shown in Table S8. As shown in this table, the best performance was obtained by the RF algorithm using the D1 features with the MAE and MAPE values of 352.91 and 14.07%, respectively. Table S9 shows that feature Subset-5 achieved the best results compared with the other subsets. The Bagging (Rep tree) method achieved the best estimation results with the MAE and MAPE values of 306.02 and 11.88%, respectively. Unlike the results obtained for other subsets, the estimation results using the random tree technique were worse than those of the MLP. Table S10 shows the experimental results obtained for Subset 6 (on D1 only because less than 40% missingness). As shown, the Bagging technique using the Rep tree achieved the best performance with the MAE and MAPE values of 356.61 and 14.18%, respectively. Further, we found that the performance of the other ML models was comparable; however, the random tree technique performed worse, showing the MAE and MAPE values of 496.18 and 18.90%, respectively.

The results obtained from the combination of all the feature subsets are represented in Table S11 which shows that best performance was achieved using LR method using D2 with a MAE and MAPE of 299.32 and 11.23%, respectively. The results after applying feature selection algorithms on combined features are shown in Table S12. The important features selected by FS techniques are: baby’s gender, gestational age at delivery of current pregnancy, blood type of mother, mother’s height, diagnosis of hypertension in mother, smoking status of mother, total antenatal visits, diagnosis of diabetes mellitus in mother, maternal age, Body Mass Index, previous pregnancy outcomes, mother’s marital status, and occupation. As shown in Table S12, the best performance was obtained using the RF algorithm, with the MAE and MAPE values of 294.53 and 11.49%, respectively. The results were improved when the feature selection technique was used compared with the results obtained from the MFE features (D2). Table S12 shows that, compared with the MFE features, the results obtained using almost all the algorithms were improved when the FS technique was used; this shows that in addition to removing irrelevant feature that aids in fast processing, the estimation results can be improved. Finally, Table 3 shows the best estimation results obtained for each feature subset. As shown in the table, the best performance was obtained using the FS technique with the RF algorithm followed by original total feature using Linear Regression with the MAE values of 294.53 and 299.32 were obtained, respectively. Among all the subsets, the best estimation results were obtained using Subset-4 with the estimation results close to the original total features set. Subset-4 achieved the best results because it contains nearly all of the relevant features selected by the FS techniques.

**Table 3:** Summary of the best results across all the subsets.

Dataset	Regression model	Original/MFE features	MAE	MAPE (%)
Subset-1	SMOReg	D2	308.98	12.13
Subset-2	Nu-SVR	D1	361.74	14.57
Subset-3	RF	D1	345.08	13.76
Subset-4	RF	D1	352.91	14.07
Subset-5	Bagging (Rep tree)	D2	306.0239	11.88
Subset-6	Bagging (Rep tree)	D1	356.61	14.18
Combined features	Linear Regression	D2	299.32	11.23
Combined features	RF	Feature selection	294.53	11.49

### LBW classification

Here, we discuss the classification performance of multiple classifiers for LBW classification. As mentioned previously, our data were highly imbalanced; therefore, to evaluate the performance of each classifier effectively, multiple performance metrics, such as accuracy, precision, recall, F-score, and a confusion matrix were used. Depending upon their application, researchers may select appropriate performance measures. Each experiment was performed using the five-fold cross validation techniques, and the results were presented as the average of all folds. Table S13 shows the performance of multiple classifiers for LBW classification for Subset-1. It can be seen from Table S13 that LR was best in all performance metrics while Bagging (NB) achieved similar performance in F1-score and confusion matrix. The results from the confusion matrix show that LR could classify 142 ABW and only 4 LBW; however, its performance is better than all the other classifiers. For example, the accuracy of the KNN technique is 89.02%, which is close to the accuracy of the LR classifier. However, the KNN technique could not classify the LBW samples; thus, its performance was poor. Similarly, the Kstar technique correctly classified 9 LBW samples, which is better than the LR classifier. However, the Kstar technique’s performance deteriorated when classifying the ABW samples. Thus, its overall performance was poor. Therefore, the best performance was obtained by the LR classifier using the MFE criterion. Similarly, the performance of the NB classifier was also improved. The results obtained using feature Subset-2 are shown in Table S14, which show that the best performance for LBW classification was obtained by the random tree technique with the accuracy, precision, recall, and F-score. We found that the random tree technique correctly classified two samples of the minority class and 132 samples of the majority class which is relatively better than other classifiers. In addition, its performance with data imputation was further reduced, and the best performance among all classifiers when data imputation was used was increased by over 2% with an accuracy of 79.05 using the random tree classifier.

The results obtained for features Subset-3 are shown in Table S15. As shown in this table, the best results were obtained using the NB classifier with an accuracy of 69.85%, which correctly classified 7 LBW samples. In addition, the kStar technique with DF correctly classified all the LBW samples; however, it was unable to classify the ABW samples. As a result, it demonstrated a poor accuracy of only 10%. However, the performance of the kStar technique using the MFE criterion resulted in an accuracy of 85.37%; however, this technique only identified 2 LBW samples. Thus, the NB and Bagging (NB) techniques achieved the best classification performance which correctly classified 7 LBW samples using the DF criterion followed by the random tree technique using the MFE criterion. With data imputation, the random tree technique performed well by classifying 127 ABW samples and 4 LBW samples with the accuracy and precision values of 79.78 and 82.2, respectively. The best performance was achieved using the Bagging (NB) classifier when features Subset-4 with DF were used. Herein, the accuracy, precision, recall, and F-score values of 82.5, 83.0, 82.5, and 0.82 were obtained, respectively (Table S16).

Table S17 shows the results obtained on features Subset-5. As shown in this table, the best performance was achieved using the LR classifier with the accuracy, precision, recall, and F-score values of 90.38, 87.5, 90.3, and 0.87, followed by the Bagging (NB) technique with the values of 89.47, 89.1, 89.4, and 0.89, respectively. It can be seen from Table S17 that LR was better because it achieved better accuracy, recall, and also performed well on confusion matrix followed by Bagging (NB) which achieved better precision, and F1-score. Table S17 shows that the LR classifier classifies the maximum number of samples, i.e., 148 correctly classified samples with 4 LBW samples, while the Bagging (NB) technique correctly classified 146 samples with 7 correctly classified LBW samples. The performance obtained using basic data imputation was reduced by ~ 2% in accuracy compared with the default experimental setting (Table S17). The Bagging (NB) technique achieved similar performance for features Subset-6, and the kStar technique performed well for this features subset, as shown in Table S18.

Finally, the performance of all the classifiers was evaluated was evaluated using all features, and the results are shown in Table S19. As shown in this table, best results were obtained using the MLP classifier, which achieved the accuracy, precision, recall, and F-score values of 88.58, 87.1, 87.9, and 0.86, respectively. Similar performance was achieved by LR classifier. The best classification results across all feature subsets are shown in Table 4. As shown in this table, the best results were obtained for features Subset-5 in all performance measures followed by the total features set. In terms of LBW sample classification, the Bagging (NB) technique with the full dataset showed the best performance. Subset-5 performed well because it contained most of the important features, as discussed in the feature selection section.

**Table 4:** Summary of the best result across all subsets.

Classifiers	Dataset	Confusion matrix			Accuracy	Precision	Recall	F1 score
Bagging (NB)	Subset-1 D2	Class	LBW	ABW	89.18	87.1	89.1	0.87
		LBW	4	14				
		ABW	4	142				
Radom tree	Subset-2 D1	LBW	2	16	81.98	80.9	81.9	0.81
		ABW	14	132				
Bagging (NB)	Subset-3 D1	LBW	7	11	69.88	81.9	69.8	0.73
		ABW	35	111				
Bagging (NB)	Subset-4 D1	LBW	4	14	82.5	83.0	83.2	82.4
		ABW	14	132				
Bagging (NB)	Subset-5 D1	LBW	**7**	**11**	**89.47**	**89.1**	**89.4**	**0.89**
		ABW	**7**	**139**				
kstar	Subset-6 D1	LBW	4	14	87.90	84.38	87.9	0.85
		ABW	6	140				
Bagging (NB)	Combined features D1	LBW	8	10	**74.56**	**83.77**	**74.55**	**0.78**
		ABW	20	126				
MLP	Combined features D1	LBW	**5**	**13**	**88.58**	**87.1**	**87.9**	**0.86**
		ABW	**6**	**140**				

Significant values are in bold.

### Data balancing using SMOTE

The results obtained when the original dataset was balanced using SMOTE with four different oversampling ratios are shown in Table S20. The results show an improved classification performance. As shown in the table, the best results were achieved using the LR classifier when the minority class was oversampled by 100%, achieving the accuracy, precision, recall, and F1-score values of 90.24, 87.6, 90.2, and 0.87, respectively. The LR classifier classified a total of 148 samples with 142 of 164 ABW samples and 6 of 18 LBW samples. We found that the results differed when the ratio of the minority sample was changed. For example, the accuracy of the LR classifier was 87.25 without SMOTE, 87.37 with 50% oversampling, 90.24 with 100% oversampling, 82.27 with 300% oversampling, and 79.35 with a fully balanced dataset. In addition, the REPTree technique correctly classified 11 LBW samples and 117 ABW samples, thereby obtaining an accuracy of 78.13% when the dataset was balanced using SMOTE. The accuracy of REPTree was also the best (86.51) when the oversampling ratio was 100%, compared with the other oversampling ratios. We also found that the performance of the NB (Bagging), NB, and MLP techniques was better without data balancing using SMOTE.

The feature selection results are shown in Table S21. As shown in this table, the MLP classifier achieved the best classification results with the accuracy, precision, recall, and F1-score values of 88.44, 86.5, 88.4, and 0.87, respectively. Herein, we found that the classification results did not improve over the original results; however, the number of features was reduced by 50% from its original size without degrading accuracy. The best overall classification results are shown in Table 5. As shown in this table, the LR classifier with 100% oversampling using SMOTE achieved the best classification performance.

**Table 5:** Summary of the best classification results.

Classifiers	Dataset (description)	Confusion matrix			Accuracy	Precision	Recall	F1 score
Bagging (NB)	D1 (Loreto Subset-1)	Class	LBW	ABW	89.47	89.1	89.4	0.89
		LBW	7	11				
		ABW	7	139				
LR	Loreto (D2 with mean, mode)	LBW	4	14	88.81	86.8	88.8	0.86
		ABW	4	142				
LR	Total Dataset (100% smote)	LBW	6	12	90.24	87.6	90.2	0.89
		ABW	4	142				
Bagging (REP)	Total Dataset (Balance)	LBW	11	7	78.13	87.3	78.1	0.81
		ABW	29	117				

## Discussion

Worldwide, one in seven babies (> 20 million) are born with LBW. This puts them at a serious risk of death, stunting, and developmental difficulties. Infant’s weight estimation prior to birth can help to reduce such incidences. Estimating and preventing LBW in infants can prevent immediate health issues. Therefore, in this study, we conducted detailed experiments for BW estimation and LBW classification using maternal features.

Our extensive experimental results (Tables S5–S21) demonstrate that the best feature subset were the features from Subset-5 (Loreto et al.^15^) for both BW estimation (Table S9) and LBW classification because this feature subset contains the most relevant features selected by the FS technique. Thus, this subset provided better results compared with the other subsets. Previous studies on BW estimation have primarily relied on the ultrasound feature because it gives more accurate results. However, in this study, we used maternal features for BW estimation because they are easy to collect without relying on the ultrasound features. The experimental results shown in Table S11 indicate that the combination of all the feature subsets afforded better estimation results compared with any single feature subset. Furthermore, the best estimation results were obtained using the RF algorithm (Table 3). We found that the FS technique improved the overall LBW classification performance and reduced the number of features from 88 to 30, which is less than 40% than its original size. The best features obtained using the FS technique were maternal diabetes, hypertension, and gestational age.

The effect of missing data was also investigated in this study. Experiments were conducted using the original features (D1) containing missing values (Table S1) and that contain missing values less than 40% (D2). The experimental results obtained using D1 and D2 for BW estimation and LBW classification show that the performance of D2 was relatively better than that of D1 with a limited number of features (88 features in D1 and 30 features in D2). These results indicate that the performance improvement was not affected if the features comprised more than 40% missing values; As such, these features were removed.

The results of our LBW classification experiments (Tables S13–S21) demonstrate that all feature subsets achieved similar performances. The best feature subset was Subset-5 (Table S17), and the worst subset was Subset-2 (Table S14). The feature Subset-2 contained only 5 features, which may not represent the whole data which may explain its poor performance. The work by Faruk et al.^12^ showed that the classification performance reported in their study was also poor, also evident in our experiments.

The data used in this study were highly class imbalanced; therefore, the SMOTE algorithm with different balancing ratios was employed to balance the data. The results (Table S20) demonstrate that the best classification performance (90.24% accuracy) was obtained when the minority class was oversampled by 100% using SMOTE with the LR classifier. Although the accuracy was high, LR could identify only 6 of the 18 LBW samples, which represents only 33% accuracy. This indicates that accuracy should not be the only performance metric, especially when the data are imbalanced. We compared all the performance measures for each algorithm. For instance, Table 5 shows that for accuracy and recall, LR achieved best performance whereas Bagging (NB) achieved best precision; the F1-score of both classifiers were the best among all the classifiers. Therefore, we conclude that for the majority of performance measures, LR performed best. In many cases (Tables S13–S21), an accuracy of 89.02% was observed during the experiment. However, the classifier was unable to classify the minority (LBW) sample indicating that the performance was poor. Other classifiers such as Zero, stacking, and SVM did not improve any feature subset. Therefore, their performance remained poor in all classification experiments.

Previous studies for BW estimation and LBW classification have used different set of features (Table 1). However, in the present study, we used a combination of all the features employed in previous studies to provide a detailed analysis. The results demonstrate that this combination of features improves the performance of BW estimation and LBW classification. We expect that this to allow both researchers and medical practitioners to focus on features that are highly relevant for BW estimation and LBW classification, helping them to take appropriate steps in a timely manner. Furthermore, our study provides a baseline to select an appropriate ML model with effective preprocessing steps and determine which ML model is good for which features that are available.

In general, our study is expected to provide a baseline for researchers working in this field to obtain promising results by selecting the most effective and efficient methods, especially for the researchers in the region with similar participant profiles. Another advantage of this study is that it can accurately predict LBW infants using small amount of data while utilizing computationally fewer complex algorithms. This work can be extended to other applications such as determining hypertensive disorders and diabetes mellitus.

The results of this study provide a considerable advantage to clinicians and researchers working in the related fields, especially within the UAE. However, some limitations must be addressed in the future research. For example, performance must be further improved and the effect of processing timing due to FS techniques must be considered to determine the time consumed owing to the irrelevant features. In this study, basic imputation techniques were used; however, in the future, we plan to include intelligent imputation techniques. Although SMOTE is very effective in terms of oversampling, in the future, other oversampling techniques, e.g., GANs, will be used. In addition, deep learning-based algorithms will be used in the future. Finally, we aim to use automated ML techniques to select the most relevant preprocessing and ML models for estimation and regression. We recommend the use of FS techniques to remove irrelevant features for improving performance and reducing computation costs. We performed five-fold cross validation testing which is standard testing approach in machine learning area. The data is collected from three hospitals. This reduces the bias due to the data. Regrading overfitting problem, we presented the testing results. The excellent accuracy of classifiers (90.24%) suggests that classifiers performed well with this relatively small dataset. Finally, Socioeconomic Status and racial differences vary in different studies. However, in this study all women are from the Emirati population. All of the Emirati population have full health insurance coverage providing them with the same level of health care at any health facility. As such, there is no difference in healthcare access between pregnant women attending these three hospitals and those who use other institutions. Therefore, this study prevents the socio-economic nuances that would affect healthcare, access to healthcare and in turn LBW classifications from being affected from the differences in nationality.

## Conclusion

In this study, we presented a comprehensive performance evaluation of multiple ML models for infant weight estimation and LBW classification using the maternal features obtained from pregnant women. For weight estimation, 10 ML models were used with different feature subsets and the combinations of subsets with and without the imputation of missing values. Moreover, important features were identified using multiple FS techniques, which aids weight estimation and LBW classification. Herein, relevant features are selected using majority voting of multiple FS techniques. In addition, a SMOTE-based data balancing technique was applied to oversample the minority class sample to realize improved classification results. The best weight estimation was obtained using the RF algorithm with an MAE value of 294.53 g, and the best classification performance was obtained using the LR and SMOTE oversampling techniques. We found that this case obtained the accuracy, precision, recall, and F1 score values of 90.24%, 87.6%, 90.2%, and 0.89, respectively. Diabetes, gestational age, and hypertension are important risk features for BW estimation and LBW classification.

## Supplementary Information

Supplementary Tables.

## Data Availability

The data presented in this study are available on request from United Arab Emirates University.
