# Supplementary material for: Infant birth weight estimation and low birth weight classification in United Arab Emirates using machine learning algorithms
Source: Sci Rep. 2022 Jul 15;12:12110. doi: 10.1038/s41598-022-14393-6 (PMC9287292; doi:10.1038/s41598-022-14393-6)
Supplement: Supplementary file 1 — Supplementary Tables. [file 41598_2022_14393_MOESM1_ESM.pdf]

Infant Birth Weight Estimation and Low Birth Weight Classification in United Arab Emirates Using Machine Learning Algorithms

Supplementary Materials

Table S. 1 Original Features obtained

Features Name	Missingness/No values (%)	Description
12 features representing the gestational age at delivery during previous pregnancies (if applicable). *The maximum number of previous pregnancies was 12.*	30 -100	Numeric<br>*indicates the number of days the pregnancy completed prior to pregnancy outcome
12 features representing the pregnancy outcome at delivery during previous pregnancies (if applicable). *The maximum number of previous pregnancies was 12.*	21 - 100	Nominal:<br>Abortion/Miscarriage<br>Cesarean<br>Vaginal<br>*indicates the pregnancy outcome of each previous pregnancy of the participant
12 features representing the birth weight of baby at delivery during previous pregnancies (if applicable). *The maximum number of previous pregnancies was 12.*	39 - 100	Numeric<br>*indicates weight of baby at pregnancy outcome
15 features representing the preterm birth status at delivery during previous pregnancies (if applicable). *The maximum number of previous pregnancies was 15.*	74 - 100	Nominal:<br>Yes, No<br>*indicates if the baby was born premature
Age of Mother	0 (2missing)	Numeric
Marital Status of Mother	0	Nominal: Married, Single
Occupation Status	11	Nominal:<br>Administrative, Employed, Housewife, Others, Student, Teacher
Education Status of Mother	3	Nominal:<br>Primary, Secondary, Secondary/HS, High School Diploma, College/University Bachelors , Masters, Doctorate
Smoking status of mother	5	Nominal:<br>Yes, No, Second Hand smoke
Number of hospital visits during current pregnancy	23	Numeric
Mother’s Blood Type	3	Nominal:<br>A, B, AB, O, Others
Parity (Living children)	27	Numeric
Mother’s Body Mass Index (kg/m^2)	35	Numeric

Mother's Height	1	Numeric
Mother's weight at conception month	13	Numeric
Mother's weight at first gestational month	82	Numeric
Mother's weight at second gestational month	68	Numeric
Mother's weight at third gestational month	62	Numeric
Mother's weight at fourth gestational month	50	Numeric
Mother's weight at fifth gestational month	46	Numeric
Mother's weight at sixth gestational month	42	Numeric
Mother's weight at seventh gestational month	34	Numeric
Mother's weight at eighth gestational month	28	Numeric
Mother's weight at ninth gestational month	29	Numeric
Gestational Diabetes Mellitus in current pregnancy	86	Nominal:<br>Yes, No
Diagnosis of Diabetes Mellitus in Mother	0	Nominal:<br>Yes, No
Diagnosis of Hypertension in Mother	0	Nominal:<br>Yes, No
Systolic blood pressure of mother at first visit in current pregnancy	7	Numeric
Diastolic blood pressure of mother at first visit in current pregnancy	7	Numeric
Systolic blood pressure of mother at first trimester in current pregnancy	46	Numeric
Diastolic blood pressure of mother at first trimester in current pregnancy	46	Numeric
Systolic blood pressure of mother at second trimester in current pregnancy	22	Numeric
Diastolic blood pressure of mother at second trimester in current pregnancy	22	Numeric
Systolic blood pressure of mother at third trimester in current pregnancy	9	Numeric
Diastolic blood pressure of mother at third trimester in current pregnancy	9	Numeric
Hemoglobin reading of mother at first trimester in current pregnancy	29	Numeric
Hemoglobin reading of mother at 2nd trimester in current pregnancy	59	Numeric
Hemoglobin reading of mother at third trimester in current pregnancy	35	Numeric
Gestational age of baby in current pregnancy	0 (3 missing values)	Numeric
Gender of baby in current pregnancy	1	Nominal:<br>Male, Female, Unknown
Birth weight of baby in current pregnancy	0	Numeric

Table S. 2 Features after removing missing values more than 30%, we rerefer this as missing features elimination (MFE) criteria or D2.

Features Name	Missingness/No values (%)	Description

Gestational age at delivery during previous pregnancies (1st pregnancy)	30	Numeric
Pregnancy outcome at delivery during previous pregnancies (1st pregnancy)	21	Nominal:
Pregnancy outcome at delivery during previous pregnancies (2nd pregnancy)	39	Nominal
Birth weight of baby at delivery during previous pregnancies (1st pregnancy)	39	Numeric
Birth weight of baby at delivery during previous pregnancies (2nd pregnancy)	49	Numeric
Age of Mother	0 (2missing)	Numeric
Marital Status of Mother	0	Nominal:
Occupation Status	11	Nominal:
Education Status of Mother	3	Nominal:
Smoking status of mother	5	Nominal:
Number of hospital visits during current pregnancy	23	Numeric
Mother's Blood Type	3	Nominal:
Parity (Living children)	27	Numeric
Mother's Body Mass Index (kg/m^2)	35	Numeric
Mother's Height	1	Numeric
Mother's weight at conception month	13	Numeric
Mother's weight at seventh gestational month	34	Numeric
Mother's weight at eighth gestational month	28	Numeric
Mother's weight at ninth gestational month	29	Numeric
Diagnosis of Diabetes Mellitus in Mother	0	Nominal:
Diagnosis of Hypertension in Mother	0	Nominal:
Systolic blood pressure of mother at first visit in current pregnancy	7	Numeric
Diastolic blood pressure of mother at first visit in current pregnancy	7	Numeric
Systolic blood pressure of mother at second trimester in current pregnancy	22	Numeric
Diastolic blood pressure of mother at second trimester in current pregnancy	22	Numeric
Systolic blood pressure of mother at third trimester in current pregnancy	9	Numeric
Diastolic blood pressure of mother at third trimester in current pregnancy	9	Numeric
Hemoglobin reading of mother at first trimester in current pregnancy	29	Numeric
Hemoglobin reading of mother at 2nd trimester in current pregnancy	35	Numeric
Gestational age of baby in current pregnancy	0 (3 missing values)	Numeric
Gender of baby in current pregnancy	1	Nominal:
Birth weight of baby in current pregnancy	0	Numeric

Table S. 3 Feature selection algorithms used in this study.

Feature selection algorithm	Description	Used for LBW classification	Used for BW estimation
Correlation-based feature selection subset evaluator [29]	Evaluates the ranks of subsets of attributes that are highly correlated to the target variable with less intercorrelation among each other.	×	×
Classifier attribute evaluator (CAE) [30]	Evaluates the rank of an attribute using a classifier in which the weight of a feature is determined by the performance without that feature.	×	×
Correlation attribute evaluator [31]	Correlation of each attribute with the target variable is calculated using Pearson's coefficient.	×	×
Gain ratio attribute evaluator [31]	Calculates the importance of an attribute with respect to the target class by calculating the gain ratio for each target class.	×	
Information gain attribute evaluator [32]	Calculates the importance of features by calculating the information gain (IG) for each target class. IG is calculated using the difference between the entropy of the class and the entropy of the given feature.	×	
OneR attribute evaluator [33]	The best feature with better classification performance is selected using the OneR classifier.	×	
Principal components [32]	Performs principal component analysis and remove the worst eigenvector so that the features with less variance are removed.	×	×
ReliefF attribute evaluator [34]	ReliefF is sixth variation from A to F proposed by Kononeko [34], and a simple but efficient method that works with noisy data [35]. It estimates relevance of attributes according to their dependency on similar attributes based on distance.	×	×
Symmetrical uncertainty attribute evaluator [33]	Evaluates the weight of each attribute by measuring the symmetrical uncertainty with respect to the class.	×	

Table S. 4 ML models used in this study for infant's BW estimation and LBW classification

ML models	Description	Used for LBW classification	Used for BW estimation
SVM	It is a well-known, supervised ML algorithm that is used for classification and regression [36, 37]. SVM can learn high-dimensional spaces with limited training samples by minimizing the bound on error and complexity. SVM separates a set of objects having different classes by finding optimal hyperplane with maximum margin between different classes. Different kernels [] are used when the data is not linearly separable.	×	×
RF	It is an ensemble of multiple decision trees where each tree has its own prediction in which each instance recursively branches left or right until the last node of decision tree is reached. All the predictions of the DT are combined by RF to afford one prediction based on majority voting or averaging [38]. RF is the ensemble of n tress $\{T_1(X), T_2(X), T_3(X), \ldots\ T_n(X)\ \}$, where X is the feature vector given to each decision tree for classification. Each tree will predict the label Y_i, where i will either be LBW or ABW. Hence, the final prediction will be $Y_{out} = \max(Y_1 = \{T_1(X), Y_2 = T_2(X), \ldots\ldots Y_n = \ T_n(X)\ \}$.	×	×
KNN	It is a simple yet efficient ML algorithm that assigns a class label $Y_i = \{\ LBW\ or\ ABW\}$ to a target instance based on retrieving its k nearest neighbors. The class label to the target instance is assigned on the basis of the majority vote among its k nearest neighbors. The distance among the nearest neighbors to the target sample can be computed using Euclidean distance, which can be computed as follows:	×	

	$d(x,y) = \sqrt{\sum_{j=1}^{d}(x_i - y_i)^2}$,<br>where x_i and y_i represent the LBW and ABW classes, respectively. An optimal value of *k* is necessary for the accurate classification of the new instance [39].		
LR	It can classify an observation into both categorical and numerical variables. Multinomial logistic regression is used when classifying an observation into more than two classes. The model is generally presented in the following format, shown in Eq. 3, where βrefers to the parameters and x represents the independent variables as shown in<br>$\log(odds) = \beta_0 + \beta_1 * x1 + \cdots + \beta_n * x_n$	×	
NB	NB classifier is a probabilistic classifier based on Bayes Theorem that predicts probabilities with an assumption that features are independent for a given class. If we have set of features $x = (x1, x2, \ldots, , xn)$ then the class can be selected using<br>$y' = argmax(P(y/x))$<br>where,<br>$P(y/x) \propto P(x/y)P(y)$.<br>Here, $P(x/y)$is the likelihood of feature for vector x and a given class y, and $P(y)$ is the priori probability of class y.	×	
Linear Regression	It assumes that there is a linear relationship between the input and target variables.		×
Sequential minimal optimization (SMO)regression [40]	It is an iterative algorithm that uses SMO with the SVM algorithm to solve the regression problems.		×
MLP [41]	MLP is an artificial neural network—inspired by human brain [42]—that employs feedforward NN with multiple layers that use backpropagation for training [41, 43]. MLP consists of multiple layers of neurons such as input, hidden layers, and output.	×	×
Gaussian processes	It is a generalized form of Gaussian probability distribution that summarizes the parameters of the functions.	×	×
Bagging [44]	It is an ensemble based ML algorithm, proposed by Breiman [44], that combines predictions of multiple generated training to form a final prediction. The combination is achieved using aggregated average for weight estimation while employing majority voting for LBW classification.	×	×
Stacking [45]	It is an ensemble serial multiclassifier that combines multiple classifiers using stacking method that are generated via different ML models on a single dataset [46].	×	
Regression by discretization	It employs a classifier on training data, where the class attributes are discretized.	×	×
Random tree [47]	It is similar to a DT; however, random tree selects the subset of predictors randomly at each node.	×	×
Reduced error pruning tree (Rep tree) [47]	It creates multiple decision trees in several iterations and selects the best tree with least prediction error that acts as a representative tree. It builds a decision tree with the highest IG value and then prunes the tree using reduced error pruning [47].	×	×
Zero rule (ZeroR)	It is a simple majority classifier that labels all the instances based on the majority class (LBW or ABW) without considering the attributes.	×	
One rule (OneR)	It generates one rule for each predictor in the dataset to predict the class and then selects the rule with the smallest possible error.	×	
Decision table [48]	It consists of a hierarchical table that takes different sets of conditions.	×	
Adaboost [49]	The Adaboost classifier is a metaestimator that fits the classifier on a dataset. Weights are then reassigned to each instance, where more weight	×	

	is assigned to the incorrectly predicted samples. Consequently, a strong classifier is created from weak classifiers.		
Kstar	This is an instance-based classifier which works with the assumption that similar instances will have similar classifications. The similarity of instances is measured using a distance function	×	

BW Estimation

Table S. 5 Results obtained for Subset-1 features using original features (D1) and MFE features (D2) (the best results are shown in bold)

Prediction algorithm	Original/MFE features	MAE	MAPE (%)
Linear Regression	D1	352.87	14.04
	D2	310.69	12.42
SMOregression	D1	357.90	14.50
	D2	308.98	12.13
MLP	D1	531.72	20.30
	D2	477.80	18.52
Gaussian Process	D1	358.16	14.19
	D2	313.96	12.44
Nu-SVR	D1	362.46	14.59
	D2	362.46	14.59
Epsilon-SVR	D1	362.50	14.69
	D2	362.50	14.69
Baggigng	D1	352.93	14.06
	D2	314.39	12.64
Random Forest	D1	349.96	13.91
	D2	314.97	12.72
Regression by Discretization	D1	376.49	14.69
	D2	342.96	13.502
Random Tree	D1	409.87	15.457
	D2	402.07	15.67
Rep tree	D1	363.75	14.49
	D2	321.82	12.89

Table S. 6 Results obtained for Subset-2 features (the best results are shown in bold)

Prediction algorithm	Mean Absolute Error	MAPE (%)
Linear Regression	364.87	14.58
SMOregression	366.64	14.84
MLP	473.39	18.13
Gaussian Process	365.94	14.63
**Nu-SVR**	**361.74**	**14.57**
Epsilon-SVR	362.50	14.65
Bagging	370.31	14.80
Random Forest	376.34	14.90
Regression by Discretization	383.90	15.23
RandomTree	413.80	16.20
Rep tree	367.66	14.72

Table S. 7 Results obtained for Subset-3 features using original features (D1) and MFE features (D2) (the best results are shown in bold)

Prediction algorithm	Original/MFE features	MAE	MAPE (%)
Linear Regression	D1	420.28	15.86
	D2	388.41	14.97
SMOregression	D1	411.65	15.78
	D2	386.57	15.09
MLP	D1	3543.22	-
	D2	437.604	17.00
Gaussian process	D1	390.1927	15.00
	D2	385.50	14.93
Nu-SVR	D1	361.477	14.62
	D2	362.05	14.57
Epsilon-SVR	D1	360.99	14.63
	D2	362.00	14.67
Bagging	D1	346.72	13.84
	D2	349.37	13.90
**RF**	D1	**345.08**	**13.76**
	D2	357.34	14.06
Regression by Discretization	D1	359.94	14.35
	D2	366.36	14.49
Random tree	D1	355.82	14.08
	D2	429.24	16.50
Rep tree	D1	355.73	14.09
	D2	356.28	14.20

Table S. 8 Results obtained for Subset-4 features using original features (D1) and MFE features (D2) (the best results are shown in bold)

Prediction algorithm	Original/MFE features	MAE	MAPE (%)
Linear Regression	D1	358.22	14.11
	D2	358.38	14.29
SMOregression	D1	359.85	14.38
	D2	359.98	14.50
MLP	D1	490.79	18.99
	D2	431.593	16.74
Gaussian process	D1	357.66	14.11
	D2	356.619	14.23
Nu-SVR	D1	362.43	14.59
	D2	362.44	14.59
Epsilon-SVR	D1	362.55	14.60
	D2	362.49	14.69
Bagging	D1	353.35	14.11
	D2	354.64	14.14
**RF**	D1	**352.91**	**14.07**
	D2	358.18	14.17
Regression by Discretization	D1	372.32	14.86
	D2	386.42	15.23
Random tree	D1	382.56	14.93
	D2	433.39	16.56
Rep tree	D1	363.72	14.48
	D2	361.87	14.41

Table S. 9 Results obtained for Subset-5 features using original features (D1) and MFE features (D2) (the best results are shown in bold)

Prediction algorithm	Original/MFE features	MAE	MAPE (%)
Linear Regression	D1	322.017	11.87
	D2	318.17	11.77
SMOregression	D1	314.8138	11.68
	D2	311.7925	11.56
MLP	D1	369.445	13.88
	D2	339.58	12.68
Gaussian Process	D1	321.494	12.11
	D2	324.0254	12.29
Nu-SVR	D1	359.7973	14.48
	D2	361.080	14.54
Epsilon-SVR	D1	359.3131	14.56
	D2	360.918	14.62
**Bagging**	D1	306.28	11.89
	**D2**	**306.0239**	**11.88**
RF	D1	309.79	12.01
	D2	314.85	12.16
Regression by Discretization	D1	332.43	12.95
	D2	323.583	12.23
Random tree	D1	350.62	13.55
	D2	404.404	14.73
Rep tree	D1	319.01	12.31
	D2	319.379	12.23

Table S. 10 Results obtained for Subset-6 features (D2) (the best results are shown in bold)

Prediction algorithm [16]	MAE	MAPE (%)
Linear Regression	381.29	14.814
SMOregression	377.85	14.93
MLP	430.31	16.50
Gaussian process	379.11	14.83
Nu-SVR	362.28	14.58
Epsilon-SVR	362.32	14.68
**Bagging (Rep tree)**	**356.61**	**14.18**
RF	362.50	14.38
Regression by Discretization	409.67	15.59
Random tree	496.18	18.90
Rep tree	364.80	14.49

Table S. 11 Results obtained for a combination of all features using original features (D1) and MFE features (D2) (the best results are shown in bold)

Prediction algorithm	Original/MFE features	MAE	MAPE (%)
**Linear Regression**	D1	356.06	13.22
	**D2**	**299.32**	**11.23**
SMOregression	D1	354.23	13.11
	D2	302.49	11.33

MLP	D1	1009.266	31.27
	D2	570.53	20.87
Gaussian process	D1	324.851	12.24
	D2	308.86	11.63
Nu-SVR	D1	362.46	14.59
	D2	362.46	14.59
Epsilon-SVR	D1	362.50	14.69
	D2	362.50	14.69
Bagging (Rep tree)	D1	305.89	11.79
	D2	306.06	11.79
RF	D1	316.69	12.61
	D2	307.65	12.10
Regression by Discretization	D1	359.81	14.38
	D2	345.0805	13.01
Random tree	D1	354.80	14.06
	D2	368.54	14.51
Rep tree	D1	327.70	12.68
	D2	327.95	12.69

Table S. 12 Results obtained using feature selection algorithms for combined features

Prediction algorithm	MAE	MAPE (%)
Linear Regression	298.25	11.21
SMOregression	306.73	11.43
MLP	421.89	15.19
Gaussian process	304.75	11.55
Nu-SVR	362.43	14.59
Epsilon-SVR	362.47	14.69
Bagging	299.50	11.54
**RF**	**294.53**	**11.49**
Regression by Discretization	341.44	12.71
Random tree	394.63	14.59
Rep tree	322.04	12.39

LBW Classification

Table S. 13 Results obtained for Subset-1 for LBW classification using original features (D1) and MFE features (D2) (the best results are shown in bold)

Classifiers	Original/MFE features	Confusion matrix			Accuracy	Precision	Recall	F1 score
ZeroR	D1	Class	LBW	ABW	89.02	-	0.89	-
		LBW	0	18				
		ABW	0	146				
	D2	LBW	0	18	89.02	-	0.89	-
		ABW	0	146				
KNN	D1	LBW	0	18	89.02	-	0.89	-
		ABW	0	146				
	D2	LBW	0	18	89.02	-	0.89	-
		ABW	0	146				
NB	D1	LBW	5	13	86.1	85.6	86.1	0.86
		ABW	10	136				
	D2	LBW	4	14	88.21	86.42	88.2	0.86
		ABW	6	140				

Bagging (NB)	D1	LBW	5	13	86.1	85.6	86.1	0.86
		ABW	10	136				
	**D2**	LBW	**4**	**14**	**89.18**	**87.1**	**89.1**	**0.87**
		ABW	**4**	**142**				
kStar	D1	LBW	9	9	53.85	81.3	52.2	0.52
		ABW	70	76				
	D2	LBW	2	16	85.05	82.1	85.1	0.83
		ABW	9	137				
MLP	D1	LBW	5	13	85.51	84.5	85.5	0.85
		ABW	11	135				
	D2	LBW	5	13				84.92
		ABW	11	135	85.54	84.53	85.54	
Random tree	D1	LBW	5	13	87.19	84.7	86.6	0.85
		ABW	8	138				
	D2	LBW	4	14	81.75	82.9	81.7	0.82
		ABW	16	130				
SVM (rbf)	D1	LBW	0	18	89.02	-	0.89	89.02
		ABW	0	146				
	D2	LBW	0	18	89.02	-	0.89	89.02
		ABW	0	146				
Adaboost	D1	LBW	3	15	85.98	84.7	86.6	0.85
		ABW	8	138				
	D2	LBW	5	13	88.61	85.2	88.6	0.88
		ABW	3	143				
LR	D1	LBW	5	13	87.9	86.5	87.8	0.86
		ABW	6	140				
	D2	LBW	**4**	**14**	**89.35**	**88.32**	**89.3**	**0.87**
		ABW	**4**	**142**				
RF		LBW	0	18	89.02	-	0.89	-
	D1	ABW	0	146				
	D2	LBW	1	17	88.43	81.9	88.4	0.84
		ABW	1	145				
OneR		LBW	2	16	88.07	85.5	88.0	0.85
	D1	ABW	4	142				
	D2	LBW	2	16	88.07	85.5	88.0	0.85
		ABW	4	142				
Rep tree		LBW	0	18	89.02	-	0.89	-
	D1	ABW	0	146				
	D2	LBW	1	17	87.59	79.78	87.59	0.83
		ABW	3	143				
Stacking (Deci Tab)		LBW	0	18	89.02	-	0.89	-
	D1	ABW	0	146				
	D2	LBW	0	18	89.02	-	0.89	-
		ABW	0	146				
Stack KNN		LBW	0	18	89.02	-	0.89	-
	D1	ABW	0	146				
	D2	LBW	0	18	89.02	-	0.89	-
		ABW	0	146				
Stack NB		LBW	0	18	89.02	-	0.89	-
	D1	ABW	0	146				
	D2	LBW	0	18	89.02	-	0.89	-
		ABW	0	146				
Stack RF		LBW	0	18	89.02	-	0.89	-
	D1	ABW	0	146				
	D2	LBW	0	18	89.02	-	0.89	-
		ABW	0	146				

SMO	D1	LBW	0	18	89.02	-	0.89	-
		ABW	0	146				
	D2	LBW	0	18	89.02	-	0.89	-
		ABW	0	146				
Decision table	D1	LBW	1	17	87.34	82.8	87.3	0.84
		ABW	4	142				
	D2	LBW	1	17	87.34	82.8	87.3	0.84
		ABW	4	142				
Bagging (REP)	D1	LBW	3	15	85.99	84.7	86.0	0.84
		ABW	8	138				
	D2	LBW	2	16	88.5	85.2	88.6	0.85
		ABW	3	143				

Table S. 14 *Results obtained for features Subset-2 for LBW classification (the best results are shown in bold)*

Classifiers	Confusion matrix			Accuracy	Precision	Recall	F1 score
Zero	Class	LBW	ABW	89.02	-	0.89	-
	LBW	0	18				
	ABW	0	146				
KNN	LBW	0	18	88.79	79.4	88.7	0.84
	ABW	1	145				
NB	LBW	0	18	89.02	-	0.89	-
	ABW	0	146				
Bagging (NB)	LBW	0	18				
	ABW	0	146	89.02	-	0.89	-
kStar	LBW	0	18	89.02	-	0.89	-
	ABW	0	146				
MLP	LBW	0	18	89.02	-	0.89	-
	ABW	0	146				
Random tree	LBW	2	16	**81.98**	**80.9**	**81.9**	**0.81**
	ABW	14	132				
SVM (rbf)	LBW	0	18	89.02	-	0.89	-
	ABW	0	146				
Adaboost	LBW	0	18	88.91	80.1	88.9	0.84
	ABW	1	145				
LR	LBW	0	18	89.02	-	0.89	-
	ABW	0	146				
RF	LBW	1	18	85.8	81.1	85.8	0.83
	ABW	7	139				
OneR	LBW	0	18	88.91	80.1	88.9	0.84
	ABW	1	145				
Rep tree	LBW	0	18	88.79	79.9	88.1	0.84
	ABW	1	145				
Stacking (Deci Tab)	LBW	0	18	89.02	-	0.89	-
	ABW	0	146				
Stack KNN	LBW	0	18				
	ABW	0	146	89.02	-	0.89	-
Stack NB	LBW	0	18	89.02	-	0.89	-
	ABW	0	146				
Stack RF	LBW	0	18	89.02	-	0.89	-
	ABW	0	146				
SMO	LBW	0	18				
	ABW	0	146	89.02	-	0.89	-
Decision table	LBW	0	18	89.02	-	0.89	-
	ABW	0	146				

Bagging (REP)	LBW	0		88.91	80.1	88.9	0.84
	ABW	1	145				

Table S. 15 Results obtained for Subset-3 for LBW classification using original features (D1) and MFE features (D2) (the best results are shown in bold)

Classifiers	Original/MFE features	Confusion matrix			Accuracy	Precision	Recall	F1 score
Zero	D1	Class	LBW	ABW	89.02	-	0.89	-
		LBW	0	18				
		ABW	0	146				
	D2	LBW	0	18	89.02	-	0.89	-
		ABW	0	146				
KNN	DF	LBW	1	17	88.56	81.5	87.8	0.82
		ABW	2	144				
	D2	LBW	0	18	89.02	-	0.89	-
		ABW	0	146				
NB	D1	LBW	7	11	**69.85**	**81.9**	**69.8**	**0.73**
		ABW	35	111				
	D2	LBW	11	7	58.7	0.64	0.58	0.59
		ABW	60	86				
Bagging (NB)	D1	LBW	7	11	**69.88**	**81.9**	**69.8**	**0.73**
		ABW	35	111				
	D2	LBW	7	11	67.51	82.3	67.6	0.71
		ABW	43	103				
kStar	D1	LBW	18	0	10.42	-	0.10	-
		ABW	146	0				
	D2	LBW	2	16	**85.37**	**81.6**	**85.12**	**0.83**
		ABW	8	138				
MLP	D1	LBW	1	17	83.03	78.8	83.0	0.81
		ABW	9	137				
	D2	LBW	1	17	85.37	81.0	85.6	0.83
		ABW	7	139				
Random tree	D1	LBW	1	17	88.56	81.5	87.8	0.82
		ABW	2	144				
	D2	LBW	2	16	83.5	0.81	82.7	0.82
		ABW	11	135				
SVM (rbf)	D1	LBW	0	18	89.02	-	0.89	89.02
		ABW	0	146				
	D2	LBW	0	18	89.02	-	0.89	89.02
		ABW	0	146				
Adaboost	D1	LBW	0	18	89.02	-	0.89	-
		ABW	0	146				
	D2	LBW	0	18	89.02	-	0.89	-
		ABW	0	146				
LR	D1	LBW	1	17	86.8	81.6	86.8	0.83
		ABW	4	142				
	D2	LBW	0	18	88.4	79.2	0.884	0.836
		ABW	1	145				
RF	D1	LBW	0	18	89.02	-	0.89	-
		ABW	0	146				
	D2	LBW	1	17	88.7	81.9	88.8	0.84
		ABW	1	145				
OneR		LBW	1	17	88.56	81.5	87.8	0.82

	D1	ABW	2	144				
	D2	LBW	0	18	89.02	-	0.89	-
		ABW	0	146				
Rep tree	D1	LBW	0	18	89.02	-	0.89	-
		ABW	0	146				
	D2	LBW	0	18	89.02	-	0.89	-
		ABW	0	146				
Stacking (Deci Tab)	D1	LBW	0	18	89.02	-	0.89	-
		ABW	0	146				
	D2	LBW	0	18	89.02	-	0.89	-
		ABW	0	146				
Stack KNN	D1	LBW	0	18	89.02	-	0.89	-
		ABW	0	146				
	D2	LBW	0	18	89.02	-	0.89	-
		ABW	0	146				
Stack NB	D1	LBW	0	18	89.02	-	0.89	-
		ABW	0	146				
	D2	LBW	0	18	89.02	-	0.89	-
		ABW	0	146				
Stack RF	D1	LBW	0	18	89.02	-	0.89	-
		ABW	0	146				
	D2	LBW	0	18	89.02	-	0.89	-
		ABW	0	146				
SMO	D1	LBW	0	18	88.4	79.2	0.884	0.836
		ABW	1	145				
	D2	LBW	0	18	88.4	79.2	0.884	0.836
		ABW	1	145				
Decision table	D1	LBW	1	17	88.56	81.5	87.8	0.82
		ABW	2	144				
	D2	LBW	0	18	89.02	-	0.89	-
		ABW	0	146				
Bagging (REP)	D1	LBW	0	18	89.02	-	0.89	-
		ABW	0	146				
	D2	LBW	1	17	89.02	0.845	0.832	0.89
		ABW	1	145				

Table S. 16 Results obtained for Subset-4 for LBW classification using original features (D1) and MFE features (D2) (the best results are shown in bold)

Classifiers	Original/MFE features	Confusion matrix			Accuracy	Precision	Recall	F1 score
Zero	D1	Class	LBW	ABW	89.02	-	0.89	-
		LBW	0	18				
		ABW	0	146				
	D2	LBW	0	18	89.02	-	0.89	-
		ABW	0	146				
	D1	LBW	0	18	89.02	-	0.89	-

KNN		ABW	0	146				
	D2	LBW	0	18	89.02	-	0.89	-
		ABW	0	146				
NB	D1	LBW	4	14	82.31	82.8	81.8	0.82
		ABW	15	131				
	D2	LBW	1		87.70	82.1	87.7	0.84
		ABW	3	143				
Bagging (NB)	D1	LBW	4	14	82.5	83.0	83.2	82.4
		ABW	14	132				
		LBW	1	17	87.69	82.1	87.7	0.83
		ABW	3	143				
kStar	D1 (linear)	LBW	13	5	33.9	80.3	33.9	0.48
		ABW	101	45				
	D2	LBW	2	16	86.44	82.1	86.4	0.84
		ABW	6	140				
MLP		LBW	2	16	82.38	80.8	82.4	0.81
		ABW	13	133				
	D2	LBW	1	17	86.95	81.0	86.9	0.84
		ABW	4	142				
Random tree		LBW	1	17	85.43	81.0	85.4	0.83
		ABW	7	139				
	D2	LBW	1	17	83.55	80.1	83.5	0.82
		ABW	10	136				
SVM (rbf)		LBW	0	18	0<br>0	18<br>146	0<br>0	18<br>146
		ABW	0	146				
	D2	LBW	0	18	0	18	0	18
		ABW	0	146				
Adaboost		LBW	0	18	89.02	-	0.89	-

		AB W	0	146				
	D2	LB W	0	18	89.02	-	0.89	-
		AB W	0	146				
LR		LB W	1	17	88.69	81.9	88.8	0.84
		AB W	1	145				
	D2	LB W	0	18	89.02	-	0.89	-
		AB W	0	146				
RF	D1	LB W	0	18	89.02	-	0.89	-
		AB W	0	146				
	D2	LB W	1	17	89.63	90.7	89.6	0.85
		AB W	0	146				
OneR	D1	LB W	1	17	88.26	86.5	87.8	0.83
		AB W	2	144				
	D2	LB W	1	17	87.8	79.2	87.0	0.83
		AB W	3	143				
Rep tree	D1	LB W	0	18	89.02	-	0.89	-
		AB W	0	146				
	D2	LB W	0	18	89.02	-	0.89	-
		AB W	0	146				
Stacking (Decision table)	D1	LB W	0	18	89.02	-	0.89	-
		AB W	0	146				
	D2	LB W	0	18	89.02	-	0.89	-
		AB W	0	146				
Stack KNN	D1	LB W	0	18	89.02	-	0.89	-
		AB W	0	146				
	D2	LB W	0	18	89.02	-	0.89	-
		AB W	0	146				
Stack NB	D1	LB W	0	18	89.02	-	0.89	-

		ABW	0	146				
	D2	LBW	0	18	89.02	-	0.89	-
		ABW	0	146				
Stack RF	D1	LBW	0	18	89.02	-	0.89	-
		ABW	0	146				
	D2	LBW	0	18	89.02	-	0.89	-
		ABW	0	146				
SMO	D1	LBW	0	18	89.02	-	0.89	-
		ABW	0	146				
	D2	LBW	0	18	89.02	-	0.89	-
		ABW	0	146				
Decision table	D1	LBW	1	17	87.8	79.2	87.0	0.83
		ABW	3	143				
	D2	LBW	0	18	87.8	79.2	87.0	0.83
		ABW	2	144				
Bagging (REP)	D1	LBW	0	18	89.02	-	0.89	-
		ABW	0	146				
	D2	LBW	0	18	89.02	-	0.89	-
		ABW	0	146				

Table S. 17 Results obtained for Subset-5 for LBW classification using original features (D1) and MFE features (D2) (the best results are shown in bold)

Classifiers	Loreto dataset	Confusion matrix			Accuracy	Precision	Recall	F1 score
Zero	D1	Class	LBW	ABW	89.02	-	0.89	-
		LBW	0	18				
		ABW	0	146				
	D2	LBW	0	18	89.02	-	0.89	-
		ABW	0	146				
KNN	D1	LBW	0	18	89.02	-	0.89	-
		ABW	0	146				
	D2	LBW	1	17	89.31	83.7	89.3	0.84
		ABW	0	146				
NB	D1	LBW	8	10	82.64	87.5	82.6	0.84
		ABW	19	127				
	D2	LBW	8	10	88.56	88.8	88.5	0.88

		ABW	9	137				
Bagging (NB)	D1	LBW	7	11	85.55	87.9	85.5	0.86
		ABW	13	133				
	D2	LBW	7	11	89.47	89.1	89.4	0.89
		ABW	7	139				
kStar	D1	LBW	1	17	88.31	83.1	88.3	0.84
		ABW	3	143				
	D2	LBW	2	16	89.16	85.41	89.16	0.85
		ABW	2	144				
MLP		LBW	3	15	89.05	87.7	89.1	0.86
		ABW	5	141				
	D2	LBW	3	15	89.77	85.86	89.7	0.86
		ABW	2	144				
Random tree		LBW	5	13	85.15	84.3	85.1	0.84
		ABW	11	135				
	D2	LBW	5	13	87.48	85.24	87.4	0.86
		ABW	8	138				
SVM (rbf)		LBW	0	18				
		ABW	0	146	89.02	-	0.89	-
	D2	LBW	0	18				
		ABW	0	146	89.02	-	0.89	-
		LBW	0	18				
		ABW	0	146	89.02	-	0.89	-
Adaboost		LBW	2	16	88.19	82.6	88.1	0.84
		ABW	3	143				
	D2	LBW	2	16	89.0	83.3	89.0	0.85
		ABW	1	145				
LR		LBW	4	14	88.81	86.8	88.8	0.86
		ABW	4	142				
	D2	LBW	4	14	90.38	87.56	90.3	0.87
		ABW	2	144				
RF		LBW	2	16	88.20	85.3	88.2	0.85
	D1	ABW	4	142				
	D2	LBW	2	16	89.16	85.31	89.1	0.85
		ABW	2	144				
OneR		LBW	2	16	88.07	85.5	88.1	0.85
	D1	ABW	4	142				
	D2	LBW	2	16	89.16	85.31	89.1	0.85
		ABW	2	144				
Rep tree		LBW	1	17	87.83	79.8	87.8	0.83
	D1	ABW	3	143				
	D2	LBW	1	17	89.16	82.78	89.1	0.85
		ABW	2	145				
Stacking (Deci Tab)		LBW	0	18				
	D1	ABW	0	146	89.02	-	0.89	-
	D2	LBW	0	18				
		ABW	0	146	89.02	-	0.89	-
Stack KNN		LBW	0	18				
	D1	ABW	0	146	89.02	-	0.89	-
	D2	LBW	0	18				
		ABW	0	146	89.02	-	0.89	-
Stack NB		LBW	0	18				
	D1	ABW	0	146	89.02	-	0.89	-
	D2	LBW	0	18				
		ABW	0	146	89.02	-	0.89	-
Stack RF		LBW	0	18				

	D1	ABW	0	146	89.02	-	0.89	-
	D2	LBW	0	18	89.02	-	0.89	-
		ABW	0	146				
SMO	D1	LBW	0	18	89.02	-	0.89	-
		ABW	0	146				
	D2	LBW	0	18	89.02	-	0.89	-
		ABW	0	146				
	Data Imptutation	LBW	0	18	89.02	-	0.89	-
		ABW	0	146				
Decision table	D1	LBW	1	17	87.83	81.8	87.8	0.83
		ABW	3	143				
		LBW	0	18	89.02	-	0.89	-
		ABW	0	146				
Bagging (REP)	D1	LBW	2	16	88.19	82.6	88.1	0.84
		ABW	3	143				
	D2	LBW	2	16	89.0	83.3	89.0	0.85
		ABW	2	144				

Table S. 18 *Results obtained for Subset-6 for LBW classification (the best results are shown in bold)*

Classifiers [16]	Confusion matrix			Accuracy	Precision	Recall	F1 score
Zero	Class	LBW	ABW	89.02	-	0.89	-
	LBW	0	18				
	ABW	0	146				
KNN	LBW	0	18	88.67	79.4	88.6	0.83
	ABW	1	146				
NB	LBW	6	12	68.67	81.78	68.67	0.71
	ABW	40	102				
Bagging (NB)	LBW	5	13	74.27	81.17	74.52	**0.76**
	ABW	28	118				
kSta	**LBW**	**4**	**14**	**87.90**	**84.38**	**87.9**	**0.85**
	ABW	6	140				
MLP	LBW	0	18	89.02	-	0.89	-
	ABW	0	146				
Random tree	LBW	6	12	85.22	85.43	85.22	0.85
	ABW	12	134				
SVM (rbf)	LBW	0	18	89.02	-	0.89	89.02
	ABW	0	146				
Adaboost	LBW	0	18	89.02	-	0.89	-
	ABW	0	146				
LR	LBW	0	18	89.02	-	0.89	-
	ABW	0	146				
RF	LBW	3	15	90.96	83.45	90.9	0.87
	ABW	1	145				
OneR	LBW	1	17	89.03	82.34	89.0	0.84
	ABW	1	145				
Rep tree	LBW	0	18	88.79	79.45	88.79	0.83
	ABW	1	145				
Stacking (Deci Tab)	LBW	0	18	89.02	-	0.89	-
	ABW	0	146				
Stack KNN	LBW	0	18	89.02	-	0.89	-
	ABW	0	146				

Stack NB	LBW	0	18	89.02	-	0.89	-
	ABW	0	146				
Stack RF	LBW	0	18	89.02	-	0.89	-
	ABW	0	146				
SMO	LBW	0	18	89.02	-	0.89	-
	ABW	0	146				
Decision table	LBW	0	18	89.02	-	0.89	-
	ABW	0	146				
Bagging (REP)	LBW	0	18	89.02	-	0.89	-
	ABW	0	146				

Table S. 19 Results obtained for a combination of all features using original features (D1) and MFE features (D2) (the best results are shown in bold)

Classifiers	Total dataset	Confusion matrix			Accuracy	Precision	Recall	F1 score
Zero	D1	Class	LBW	ABW	89.02	-	0.89	-
		LBW	0	18				
		ABW	0	146				
	D2	LBW	0	18	89.02	-	0.89	-
		ABW	0	146				
KNN	D1	LBW	1	17	89.6	83.7	88.7	0.83
		ABW	0	146				
	D2	LBW	0	18	89.02	-	0.89	-
		ABW	0	146				
NB	D1	LBW	8	10	76.2	83.7	74.55	0.77
		ABW	29	117				
	D2	LBW	10	8	76.32	87.2	76.3	0.80
		ABW	30	116				
Bagging (NB)	D1	LBW	**8**	**10**	**74.56**	**83.77**	**74.55**	**0.78**
		ABW	**20**	**126**				
	D2	LBW	10	8	75.72	87.3	0.79.4	0.82
		ABW	32	114				
kStar	D1 (linear)	LBW	18	0	10.42	-	0.10	-
		ABW	146	0				
	D2	LBW	2	15	81.75	81.10	81.72	0.81
		ABW	15	132				
MLP	D1	LBW	5	13	**88.58**	**87.1**	**87.9**	**0.86**
		ABW	6	140				
	D2	LBW	5	13	86.03	85.22	86.02	0.855
		ABW	10	136				
Random tree	D1	LBW	2	16	86.25	81.3	67.2	0.83
		ABW	5	141				
	D2	LBW	4	14	84.96	84.5	84.9	0.84
		ABW	10	136				
SVM (rbf)	D1	LBW	0	18	89.02	-	0.89	-
		ABW	0	146				
	D2	LBW	0	18	89.02	-	0.89	-
		ABW	0	146				
Adaboost	D1	LBW	3	15	89.0	80.2	0.89	0.84
		ABW	1	145				
	D2	LBW	3	15	86.17	84.2	86.17	0.84
		ABW	7	139				

LR	D1	LBW	5	13	87.69	86.32	87.7	0.86
		ABW	6	140				
	D2	LBW	5	13	87.25	86.5	87.2	0.86
		ABW	8	138				
RF	D1	LBW	0	18	89.02	-	0.89	-
		ABW	0	146				
	D2	LBW	0	18	88.4	79.2	88.4	0.83
		ABW	1	145				
OneR	D1	LBW	2	16	88.5	86.6	88.6	0.85
		ABW	2	144				
	D2	LBW	2	16	87.5	84.1	87.5	0.85
		ABW	5	141				
Rep tree	D1	LBW	2	16	88.8	85.5	88.1	0.85
		ABW	1	145				
	D2	LBW	3	15	86.9	85.16	86.2	0.85
		ABW	6	140				
Stacking (Deci Tab)	D1	LBW	0	18	89.02	-	0.89	-
		ABW	0	146				
	D2	LBW	0	18	89.02	-	0.89	-
		ABW	0	146				
Stack KNN	D1	LBW	0	18	89.02	-	0.89	-
		ABW	0	146				
	D2	LBW	0	18	89.02	-	0.89	-
		ABW	0	146				
Stack NB	D1	LBW	0	18	89.02	-	0.89	-
		ABW	0	146				
	D2	LBW	0	18	89.02	-	0.89	-
		ABW	0	146				
Stack RF	D1	LBW	0	18	89.02	-	0.89	-
		ABW	0	146				
	D2	LBW	0	18	89.02	-	0.89	-
		ABW	0	146				
SMO	D1	LBW	0	18	88.4	79.2	0.884	0.836
		ABW	1	145				
	D2	LBW	0	18	89.02	-	0.89	-
		ABW	0	146				
Decision table	D1	LBW	1	17	88.42	82.23	88.4	0.84
		ABW	2	144				
	D2	LBW	1	16	85.1	82.3	85.2	0.83
		ABW	8	139				
Bagging (REP)	D1	LBW	2	16	88.7	83.97	88.8	0.85
		ABW	2	144				
	D2	LBW	3	15	86.17	84.2	85.0	0.84
		ABW	7	139				

3.1. Data Balancing Using SMOTE

Table S. 20 Results obtain for a combination of all features MFE features (D2) with different oversampling ratio (the best results are shown in bold)

Classifiers	Oversampling ratio of minority class	Confusion matrix dataset Total	Accuracy	Precision	Recall	F1 score

Zero	50%	Class	LBW	AB W	89.02	-	0.89	-
		LBW	0	18				
		ABW	0	146				
	100	LBW	0	18	89.02	-	0.89	-
		ABW	0	146				
	300	LBW	0	18	89.02	-	0.89	-
		ABW	0	146				
	balance	LBW	18	0	10.83	-	1.1	-
		ABW	146	0				
KNN	50	LBW	1	17	86.49	81.2	86.4	0.83
		ABW	5	141				
	100	LBW	2	16	83.58	81.9	83.5	0.82
		ABW	11	135				
	300	LBW	4	14	75.4	81.0	75.4	0.77
		ABW	26	120				
	Balance	LBW	5	13	72.25	81.5	72.2	0.76
		ABW	33	113				
NB	50	LBW	11	7	61.96	86.3	61.9	0.68
		ABW	56	90				
	100	LBW	12	6	60.76	86.8	60.7	0.67
		ABW	59	87				
	300	LBW	10	8	57.69	83.9	57.6	0.64
		ABW	62	84				
	Balance	LBW	10	8	56.71	82.7	56.7	0.63
		ABW	63	83				
Bagging (NB)	50	LBW	9	9	71.95	85.9	71.9	0.76
		ABW	37	109				
	100	LBW	9	9	68.76	85.1	68.7	0.74
		ABW	43	103				
	300	LBW	9	9	63.41	83.4	63.4	0.69
		ABW	51	95				
	Balance	LBW	9	9	61.33	82.8	61.3	0.67
		ABW	55	91				
kStar	50	LBW	4	14	82.83	83.8	82.8	0.83
		ABW	15	131				
	100	LBW	6	12	80.64	83.9	80.6	0.82
		ABW	20	126				
	300	LBW	7	11	73.69	83.0	73.6	0.77
		ABW	32	114				
	Balance	LBW	9	9	67.74	84.2	67.7	0.73
		ABW	45	101				
MLP	50	LBW	5	13	83.83	85.2	83.8	0.84
		ABW	11	135				
	100	LBW	5	13				0.84
		ABW	12	134	84.81	85.1	84.8	
	300	LBW	5	13	82.86	84.3	82.8	0.83
		ABW	15	131				
	Balance	LBW	6	12	82.74	84.9	82.7	0.83
		ABW	16	130				
Random tree	50	LBW	3	15	78.10	80.8	78.1	0.79
		ABW	21	125				
	100	LBW	5	13	82.24	83.9	82.2	0.83
		ABW	16	130				
	300	LBW	5	13	81.15	83.8	81.1	0.82
		ABW	18	128				

	Balance	LBW	7	11	77.01	83.9	77.0	0.79
		ABW	27	119				
SVM (rbf)	50	LBW	0	18	89.02	-	0.89	-
		ABW	0	146				
	100	LBW	0	18	89.02	-	0.89	-
		ABW	0	146				
	300	LBW	0	18	89.02	-	0.89	-
		ABW	0	146				
	Balance	LBW	0	18	88.41	79.2	88.4	0.83
		ABW	1	145				
Adaboost	50	LBW	5	13	85.91	86.8	85.9	0.85
		ABW	10	136				
	100	LBW	5	13	86.51	86.9	86.5	0.86
		ABW	7	139				
	300	LBW	8	10	82.76	86.9	82.7	0.84
		ABW	19	127				
	Balance	LBW	11	7	78.13	87.3	87.1	0.81
		ABW	29	117				
LR	50	LBW	6	12	87.37	87.5	87.3	0.87
		ABW	9	137				
	100	LBW	**6**	**12**	**90.24**	**87.6**	**90.2**	**0.89**
		ABW	**4**	**142**				
	300	LBW	7	11	82.27	85.9	82.2	0.83
		ABW	18	128				
	Balance	LBW	8	10	79.35	86.0	79.3	0.81
		ABW	24	122				
RF	50	LBW	1	17	87.95	85.5	89.9	0.84
		ABW	3	143				
	100	LBW	3	15	89.05	87.2	89.0	0.86
		ABW	3	143				
	300	LBW	4	14	87.72	86.9	87.7	0.86
		ABW	7	139				
	Balance	LBW	4	14	86.02	85.7	86.0	0.85
		ABW	9	137				
OneR	50	LBW	3	15	79.66	82.0	79.6	0.79
		ABW	19	127				
	100	LBW	3	15	84.15	85.3	84.1	0.83
		ABW	11	135				
	300	LBW	1	17	86.61	82.1	86.6	0.83
		ABW	6	140				
	Balance	LBW	2	16	84.05	81.0	84.0	0.82
		ABW	10	136				
Rep tree	50	LBW	5	13	84.57	86.1	84.5	0.84
		ABW	13	133				
	100	LBW	4	14	84.21	84.9	84.2	0.83
		ABW	13	133				
	300	LBW	5	13	82.40	85.5	82.4	0.83
		ABW	17	129				
	Balance	LBW	7	11	81.41	86.1	81.4	0.83
		ABW	20	126				
Stacking (Deci Tab)	50	LBW	0	18	89.02	-	0.89	-
		ABW	0	148				
	100	LBW	0	18	89.02	-	0.89	-
		ABW	0	148				
	300	LBW	0	18	89.02	-	0.89	-
		ABW	0	148				

	Balance	LBW	14	4	26.50	16.8	26.5	0.18
		ABW	117	29				
Stack KNN	50	LBW	0	18	89.02	-	0.89	-
		ABW	0	148				
	100	LBW	0	18	89.02	-	0.89	-
		ABW	0	148				
	300	LBW	0	18	89.02	-	0.89	-
		ABW	0	148				
	Balance	LBW	18	0				
		ABW	146	0				
Stack NB	50	LBW	0	18	89.02	-	0.89	-
		ABW	0	148				
	100	LBW	0	18	89.02	-	0.89	-
		ABW	0	148				
	300	LBW	0	18	89.02	-	0.89	-
		ABW	0	148				
	Balance	LBW	0	18	89.02	-	0.89	-
		ABW	0	148				
Stack RF	50	LBW	0	18	89.02	-	0.89	-
		ABW	0	148				
	100	LBW	0	18	89.02	-	0.89	-
		ABW	0	148				
	300	LBW	0	18	89.02	-	0.89	-
		ABW	0	148				
	Balance	LBW	14	4	26.50	16.8	26.5	0.18
		ABW	117	29				
SMO	50	LBW	0	18	88.55	79.43	88.5	0.84
		ABW	1	145				
	100	LBW	3	15	88.09	85.3	88.1	0.85
		ABW	4	142				
	300	LBW	5	13	81.90	84.3	89.9	0.82
		ABW	17	129				
	Balance	LBW	6	12	78.02	84.3	78.0	0.80
		ABW	24	122				
Decision table	50	LBW	2	16	87.95	83.2	87.9	0.85
		ABW	4	142				
	100	LBW	5	13	84.57	85.6	84.5	0.84
		ABW	13	133				
	300	LBW	6	12	76.77	84.0	76.7	0.79
		ABW	27	119				
	Balance	LBW	7	11	71.53	82.8	71.7	0.75
		ABW	36	110				
Bagging (REP)	50	LBW	5	13	85.91	86.85	86.9	0.85
		ABW	10	136				
	100	LBW	5	13	86.51	86.9	86.5	0.86
		ABW	7	139				
	300	LBW	8	10	82.76	86.9	82.7	0.84
		ABW	19	127				
	Balance	LBW	**11**	**7**	**78.13**	**87.3**	**78.1**	**0.81**
		ABW	**29**	**117**				

Table S. 21 Results obtained using feature selection algorithms for a combination of all features using MFE features (D2) (the best results are shown in bold)

Classifiers [15]	Confusion matrix			Accuracy	Precision	Recall	F1 score
Zero	Class	LBW	ABW	89.02	-	0.89	-
	LBW	0	18				
	ABW	0	146				
KNN	LBW	0	18	89.02	-	0.89	-
	ABW	0	146				
NB	LBW	8	10	82.63	87.2	82.6	0.84
	ABW	19	127				
**Bagging (NB)**	LBW	**8**	**10**	**83.37**	**87.5**	**83.7**	**0.84**
	ABW	**17**	**129**				
kSta	LBW	2	16	84.78	82.3	84.7	0.83
	ABW	9	137				
**MLP**	LBW	**5**	**13**	**88.44**	**86.5**	**88.4**	**0.87**
	ABW	**6**	**140**				
Random tree	LBW	4	14	84.42	83.7	84.4	0.84
	ABW	12	6				
SVM (rbf)	LBW	0	18	89.02	-	0.89	-
	ABW	0	146				
Adaboost	LBW	3	15	87.60	86.5	87.6	0.85
	ABW	6	140				
LR	LBW	4	14	88.46	86.4	88.4	0.86
	ABW	5	141				
RF	LBW	2	16	88.68	85.7	88.6	0.85
	ABW	2	144				
OneR	LBW	2	16	88.07	85.5	88.1	0.85
	ABW	4	142				
Rep tree	LBW	1	17	87.95	82.2	87.9	0.84
	ABW	3	143				
Stacking (Deci Tab)	LBW	0	18	89.02	-	0.89	-
	ABW	0	146				
Stack KNN	LBW	0	18				
	ABW	0	146	89.02	-	0.89	-
Stack NB	LBW	0	18	89.02	-	0.89	-
	ABW	0	146				
Stack RF	LBW	0	18	89.02	-	0.89	-
	ABW	0	146				
SMO	LBW	0	18	89.02	-	0.89	-
	ABW	0	146				
Decision table	LBW	1	17	87.58	82.8	87.5	0.84
	ABW	3	143				
Bagging (REP)	LBW	3	15	87.60	86.5	87.6	0.85
	ABW	6	142				
